# Current Trends and Challenges of Microbiome Research in Bladder Cancer

**DOI:** 10.1007/s11912-024-01508-7

**Published:** 2024-02-20

**Authors:** Ilaha Isali, Emma K. Helstrom, Nicole Uzzo, Ankita Lakshmanan, Devika Nandwana, Henkel Valentine, Mohit Sindhani, Philip Abbosh, Laura Bukavina

**Affiliations:** 1 Department of Urology, University Hospitals Cleveland Medical Center, Cleveland, OH USA; 2 Case Western Reserve School of Medicine, Cleveland, OH USA; 3 Department of Urology, Fox Chase Cancer Center, Philadelphia, PA USA

**Keywords:** Microbiome, Bladder cancer, Urine microbiome, Gut microbiome, Urothelial carcinoma, 16S

## Abstract

**Purpose of the Review:**

Microbiome research has provided valuable insights into the associations between microbial communities and bladder cancer. However, this field faces significant challenges that hinder the interpretation, generalization, and translation of findings into clinical practice. This review aims to elucidate these challenges and highlight the importance of addressing them for the advancement of microbiome research in bladder cancer.

**Recent Findings:**

Recent findings underscore the complexities involved in microbiome research, particularly in the context of bladder cancer. Challenges include low microbial biomass in urine samples, potential contamination issues during collection and processing, variability in sequencing methods and primer selection, and the difficulty of establishing causality between microbiota and bladder cancer. Studies have shown the impact of sample storage conditions and DNA isolation kits on microbiome analysis, emphasizing the need for standardization. Additionally, variations in urine collection methods can introduce contamination and affect results. The choice of 16S rRNA gene amplicon sequencing or shotgun metagenomic sequencing introduces technical challenges, including primer selection and sequencing read length. Establishing causality between the microbiota and bladder cancer requires experimental methods like fecal microbiota transplantation and human microbiota-associated murine models, which face their own set of challenges. Translating microbiome research into therapeutic applications is hindered by methodological variability, incomplete understanding of bioactive molecules, imperfect animal models, and the inherent heterogeneity of microbiome communities among individuals.

**Summary:**

Microbiome research in bladder cancer presents significant challenges stemming from technical and conceptual complexities. Addressing these challenges through standardization, improved experimental models, and advanced analytical approaches is essential for advancing our understanding of the microbiome’s role in bladder cancer and its potential clinical applications. Achieving this goal can lead to improved patient outcomes and novel therapeutic strategies in the future.

## Introduction

Studying the microbiome presents numerous challenges, primarily originating from the complex dynamics of microbial communities and their interactions with human hosts [1]. These challenges include variability in microbiome composition, difficulty in determining causality, challenges in integrating microbiome research into clinical practice [2]. While widespread mapping has led to the identification of the associations, correlations, and prediction between the microbiome and various health outcomes, including bladder cancer, these substantial advances also came the recognition of major technical and conceptual obstacles challenging interpretation, generalization, and translation of microbiome findings to clinical bedside.

In this review, we will delve into the challenges of interpreting microbiome research in bladder cancer. We will explore how the considerable variability in sample collection, processing, and analytical methodologies across studies complicates our understanding. Additionally, the manuscript will address the impact of contaminations and annotation errors at each stage of microbiome processing and analysis, which introduce artifacts and biases. These complexities make it challenging to distinguish true biological signals from spurious results, especially in tissues with low or non-existent microbial abundance such as urine. We will emphasize the importance of recognizing the scope, limitations, and confounders inherent in these processes. Our discussion will also cover the necessity of better harmonizing these methodologies, coupled with the inclusion of comprehensive technical and biological controls, to achieve more accurate, generalizable, and reproducible interpretations applicable across diverse populations, geographies, genders, and ethnicities. However, we will also consider the potential drawbacks of excessive standardization in microbiome processing and analysis, which might limit technological variability and diversity, crucial drivers of research innovation.

### Urine Biomass

Compared to fecal microbial burden, the urinary microbiota is characterized by a relatively low biomass, with less than approximately 10^5^ colony-forming units per milliliter [3] as compared to 10^11^ bacteria per gram [4]. Given its close location to other bacterial niches with higher microbial densities, such as the gut and vagina, it is crucial to take extreme precautions to avoid the introduction of contamination during sample collection, processing, and data analysis [5]. DNA contamination poses a considerable challenge in the study of low-biomass urinary microbiota and may lead to an overestimation of particular taxa [6•]. Contaminants can originate from various sources, and the inclusion of DNA extraction blanks and non-template controls is critical for the accuracy of the study. It should be noted that using multiple sequencing replicates helps eliminate errors, and complex algorithms are being developed to denoise sequencing data [7].

### Urine Storage and DNA Kits

Standardized conditions for collection, preservation, and storage of urine for microbiome research have not been established. Analysis of the human microbiome is complex, with many layers that could decrease reproducibility due to variation between collection and storage methods across research laboratories and institutions, including urine. There have been several studies examining storage conditions of stool specimens with preservatives having varying effects depending on which one was used, with consensus of the studies that storing at colder temperatures for shorter time period is preferred [8, 9].

A study by *Jung *et al*.* [10] evaluated microbiome differences based on assay, use of preservative, and time/temperature reproducibility. Their study explores optimal conditions for preserving and analyzing the urinary microbiome in research. It focuses on the effects of a preservative (AssayAssure®), storage time, and temperature on urine samples. The authors conclude that using AssayAssure®, shorter storage times, and colder temperatures are more favorable for maintaining the integrity of the urinary microbiome. Diversity in urine microbiome samples was observed to increase when stored at room temperature for up to 3 h. However, this increase was not significant when samples were stored at 4 °C or − 20 °C. This finding highlights the importance of the time elapsed from sample collection to preparation in ensuring consistent results in urinary microbiome studies.

Furthermore, question of DNA isolation kits and overall composition differences between taxa was further evaluated by Kastens et al. [11]. They tested kits from UltraClean, Promega, PowerSoil, Qiagen Blood & Tissue, and BiOstic. Although different kits produced varying total DNA concentrations, they all reproduced similar 16S-specific sequence depths (Kruskal–Wallis, *p* = 0.806). There were no significant differences in alpha and beta diversity (Bray–Curtis) and nonmetric multi-dimensional scaling (PERMANOVA *p* = 0.87). The detection of Gram-positive versus Gram-negative bacteria was similar across tests, except the Promega kit had fewer Gram-positive bacteria.

As outlined by UROBIOME research practices [12] guidelines for urinary microbiome research, the use of either BD Vacutainer Plus (“gray top”) tubes or the addition of AssayAssure (nucleic acid stabilizer) directly to the samples in a 1:10 ratio is recommended for culture-based and culture-independent analysis, respectively. These recommendations further highlight that standardizing these conditions is crucial for the reproducibility of microbiome studies and this is particularly relevant for urinary microbiome research, where the preservation of low biomass samples is a significant concern.

### Urine Collection

Research on urinary tract microbiota faces unique challenges, primarily due to the low biomass of urine, which increases the risk of contamination and technical biases. Addressing these issues requires meticulous specimen collection, handling, and bioinformatic processing. The method of urine collection is crucial in study design, as different techniques can introduce varying biological contaminants. Techniques include midstream voiding, urethral catheterization, suprapubic aspiration, or cystoscope evacuation. Voided urine samples are prone to contamination from urethral, genital, and dermal microbiota. In contrast, methods like cystocentesis and urethral catheterization are believed to more accurately represent the bladder’s microbiota. A recent consensus suggests distinct terminology: “urinary bladder” for samples obtained directly from the bladder (via urethral catheterization, cystocentesis, or cystoscopic collection) and “urogenital” for voided samples. This distinction helps in accurately categorizing and studying urinary tract microbiota [12].

In humans, the collection methodology and influence of microbial burden has been a hot topic of discussion, with early studies implying equivalence among sample collection [13] methodologies. However, subsequent analysis both by *Pohl *et al. [14] and *Bukavina *et al. [15] highlighted critical differences, particularly due to urethral, prostatic, vaginal and skin contamination, with catheterized samples demonstrating lower abundance of *Lactobacillus*, *Cutibacterium*, and *Corynebacterium.* Notably, most differences between bladder cancer patients and healthy controls were influenced more by collection methods than the presence of cancer. This underscores the importance of understanding collection methodologies in studies, as inaccurate cross-comparison can lead to misrepresented results.

With the contamination present within the urogenital tract, additional evaluation of possible decontamination bioinformatics and ability to apply decontamination to voided samples has been evaluated. The study conducted by *Mueller *et al*.* centered on the challenge of purifying voided urine samples by removing the influence of vulvovaginal microbiota. This was done in an effort to accurately characterize the urinary microbiome. They aimed to determine if eliminating certain vaginal taxa from the analysis could render the microbiome of voided samples comparable to that obtained from catheterized samples. However, their findings revealed that no specific level of vaginal taxa removal could achieve this equivalence. In other words, this suggests that the methods used for decontaminating voided urine samples are not sufficient to accurately represent the microbiome of samples obtained directly from the bladder.

## Urine Volume

When collecting urine for microbiome analysis, a crucial factor is the volume of urine required for adequate bacterial DNA quantification. The microbial burden in voided versus catheterized urine plays a significant role in this. In the case of voided urine, which typically has a higher level of microbial contamination, studies have shown that extracting bacterial DNA from as little as 1–2 ml of urine can be 85% effective [16]. On the other hand, urine collected via catheter generally contains a lower microbial biomass. To effectively extract and amplify bacterial DNA from such samples, larger volumes are recommended. Although the precise volume needed to yield a substantial amount of bacterial DNA is not definitively known, volumes of 30–50 ml are advised for catheter-collected urine, based on the success of similar approaches in previous research involving bladder urinary and genitourinary microbiota studies [11, 15].

### Technical Challenges

Upon extracting DNA, researchers must choose between 16S rRNA gene amplicon sequencing, which targets specific variable regions for microbial discrimination, or shotgun metagenomic sequencing, which fragments and reads entire microbial genomes from patient samples [17]. While 16S profiling is the most popular approach used to study microbial diversity, including in urine, it is flanked by several limitations, including primer selection (V1-V9). Research on the selection of 16S primers has revealed notable variations in species richness and diversity depending on the primer used. Investigations have consistently found that using only the V3 primer tends to underestimate species Richness [18]. On the other hand, while the V4 primer provides estimates that align more closely with full-length 16S sequencing and mirrors community profiles seen in shotgun sequencing, most studies primarily focus on specific variable regions. These include either the single V4 region, following the standardized protocol of the Earth Microbiome Project (EMP) [19], or the combined V1–V3 and V3–V5 regions, as per the dual-indexing protocol of the Human Microbiome Project (HMP) [20]. This preference is largely due to the limitations of the widely utilized Illumina sequencing platform, which produces only short sequences (with NextSeq, MiniSeq, iSeq producing sequences ≤ 300 bases, and MiSeq ≤ 600 bases). Recent research has consistently demonstrated that the frequently used V4 sub-region of the 16S rRNA gene is the least accurate in identifying taxa typically found in the human body. Additionally, this region, along with the V3–V5 region, is especially prone to unintended amplification of human DNA. In other words, the use of primers can amplify human DNA resulting in false positive results, with potential loss of rare taxa. A study by Heidrich et al. assessed choice of 16S rRNA primers in male urinary microbiota profiling, noting V1V2 as more suitable for urinary sampling, providing highest number of exclusively detected genera [21•]. One might consider that merging V1-V6 amplicons results in a significant rise in the variety of species identified; however, this was not shown. This suggests that the use of all V1-V6 primers may not justify the increased expenses associated with sequencing libraries containing multiple amplicons and may lead to overestimation of taxonomic richness due to the presence of falsely identified taxa [21•].

When using 16S amplicon sequencing with Illumina’s paired-end 250-bp chemistry, a choice must be made between sequencing longer segments that cover multiple variable regions of the 16S rRNA gene (such as V1-V2) and opting for shorter segments (like V4). The longer segments provide more sequence information, which is beneficial for taxonomic classification in downstream analyses. However, the quality of the sequence reads tends to deteriorate towards the ends. In contrast, with shorter segments, the problem of declining quality is mitigated because the reads from both directions overlap, allowing sequencing errors to be identified and corrected by comparing the complementary reads. For longer segments, the overlap of lower-quality sequences in the middle can lead to the creation of artifacts, falsely inflating the perceived diversity of the sample. In other words, while longer primers may provide higher Richness, these results have been questioned as false positives thus shorter regions are preferred for accurate representation by UROBIOME guidelines [12].

## Determining Causality

**Fig. 1:**
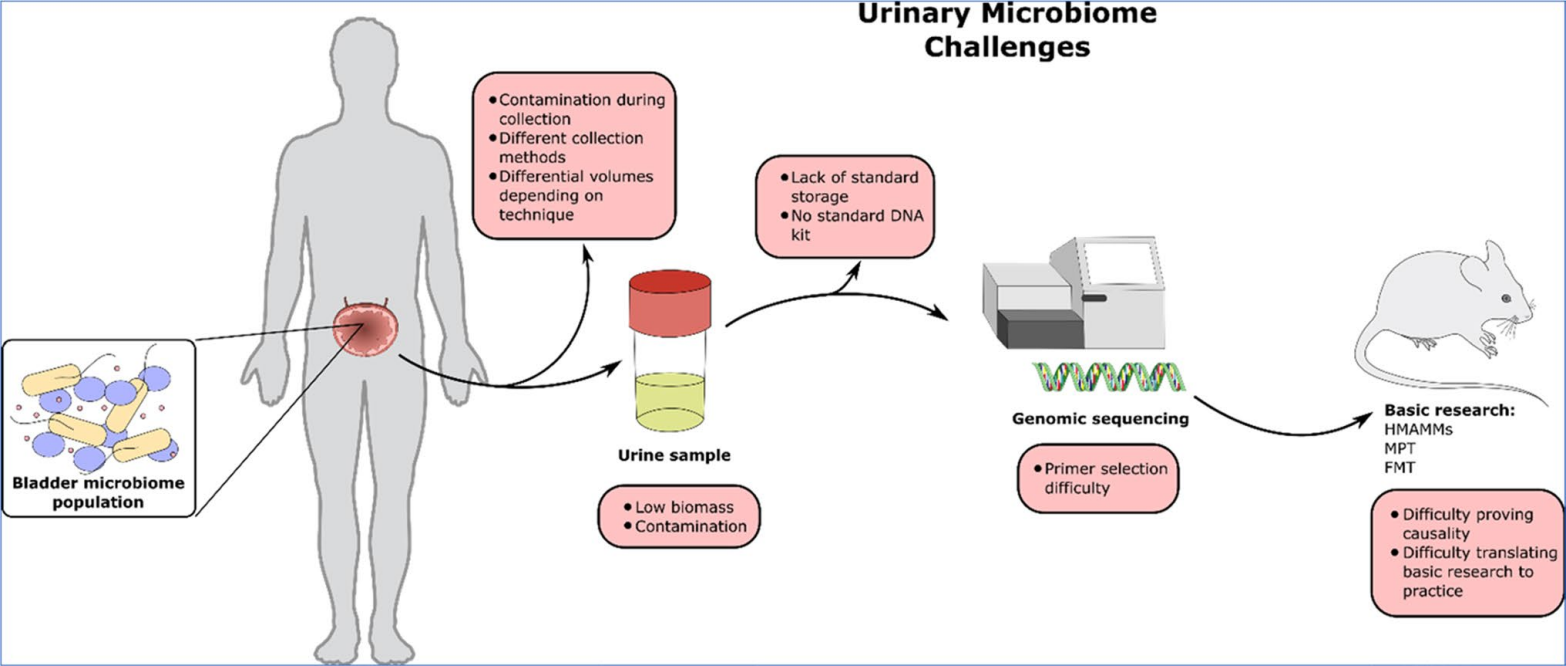
Challenges in Bladder Cancer Microbiome Research: An overview of methodological difficulties in the study of the urinary microbiome: Issues include potential contamination during sample collection, variable sample storage conditions affecting microbial DNA integrity, difficulties in primer selection for low biomass samples, and the challenge of translating research findings into clinical practice, as represented by the laboratory-to-mouse transition

Although connections between particular microorganisms or microbial patterns and BC have been noted, proving a direct causative relationship is still challenging to demonstrate [22]. Detecting enduring patterns is further complicated by the microbiome’s inherent diversity, which occurs between individuals due to genetics, epigenetics, and environmental factors. The safety and effectiveness of these therapies in BC patients are yet unknown [23]. At the same time, the medical/research community investigates the possibility of modifying the microbiome for therapeutic purposes, such as through probiotics or fecal transplants [24]. Additionally, integrating these data with other types, such as genomic and metabolomic data, is essential to fully comprehend the microbiome’s role in BC, which is crucial [25••, 26]. Lastly, even if specific microbial patterns are identified in connection with BC, determining their clinical significance for diagnosis, prognosis, or treatment involves additional complexity (Fig. 1) [26].

There are several experimental methods for inferring causality between the microbiota and diseases, such as fecal microbiota transplantation (FMT), human microbiota-associated murine models (HMAMMs), and microbe-phenotype triangulation (MPT) [27, 28]. Briefly, FMT is medical procedure where fecal matter is collected from a tested and healthy donor and then transferred to a patient, usually through a colonoscopy, endoscopy, enema, or in capsule form. HMAMMs are research models where the gut microbiota of humans is transferred into mice. This process allows researchers to study the human gut microbiome in a controlled environment, observing how it interacts with the host (in this case, the mouse) and influences health and disease. Lastly, the MPT process involves analyzing data from different studies and sources to find recurring associations between certain microbes and specific health conditions or disease states. By “triangulating” these associations from multiple angles — such as genetic, biochemical, and observational studies — researchers can more confidently infer a link between certain microbes and specific phenotype [29]*.* Challenges in experimental approaches for establishing causality include the risk of serious infections associated with FMT, as well as the significant disparities between humans and mice, which make it difficult to accurately replicate microbial communities from humans in mouse models. In contrast to numerous human diseases where microbiome research has been conducted on thousands of patients, such as in the Human Microbiome Project, the study of bladder cancer and its relation to the urine microbiome is relatively new and has been carried out with a limited number of samples. Consequently, the application of microbiome genome-wide association studies (mGWAS) [30] and other computational methods in this area is restricted due to the smaller sample size.

### Difficulties in Translating Microbiome Research to Therapeutic Applications

A significant barrier to the translation of microbiome research to clinical application is the variability in methodologies employed. Both 16S rRNA gene sequencing and WGS techniques have limitations that are greatly slowing the progression of basic research to therapeutic application. Despite being more cost effective and offering large databases of data archives, 16S sequencing has been found to be less robust than WGS. In a comparison between the two methods, Ranjan et al. found that the 16S method identified half as many species compared to WGS as well as offering inferior Shannon diversity, Simpson diversity, and evenness [31]. 16S sequencing results can be highly variable and impacted by preservatives, sample storage methods, and differences in lysis and PCR protocols. In an evaluation of forty 16S rRNA sequencing studies on microbiome associations with various cancers, Manzoor et al. noted that 26 of these studies lacked the necessary information to perform a replicate analysis. Additionally, there is no consistent option for third-party access to data, with some articles depositing data into different databases and others only making data available upon request [32]. Furthermore, the method of microbiome analysis using urine samples to understand bladder cancer is itself not without flaws. It has been previously shown by Eckburg et al. that fecal microbiome varies from the microbiome of intestinal mucosa [33]. A similar trend may exist between the bladder and urine samples. Without a reliable and repeatable methodology for finding and consolidating results of microbiome research, it will be impossible to generate the comprehensive understanding necessary for its translation into treatments.

The translation of microbiome research into clinical applications is further challenged by the incomplete understanding of bioactive molecules derived from microbiome species. With the great complexity of the microbiome, consideration of up and downregulated microbiota alone will most likely not result in novel cancer treatments as this type of research cannot elucidate the mechanisms of action. Microbiota-derived bioactive molecules may interact with multiple cellular receptors or induce a variety of immune responses [34]. A significant portion of current microbiome research demonstrates correlative and associative properties between microbiota and diseases such as cancer, and a significant effort is currently directed towards establishing causal relationships. More advanced in vivo models that allow for greater control are under development for this task [35]. A thorough understanding of these mechanisms is necessary for the safe and effective application of microbiome research in human trials.

An additional barrier to establishing causality in microbiome research is the imperfect representation of the human microbiome in animal models. The human microbiome can differ greatly from that of the animals used during in vivo testing. Zhang et al. confirmed this observation in a gut microbiome analysis, where it was found that rodent models have unique microbiota not present in humans and some human microbiota will not colonize in animal models [36].

A final challenge facing the application of microbiome research in a clinical setting is the overall heterogeneity of microbiome communities. With each person having unique microbiota, it can be very difficult to generalize findings from studies with relatively limited sample populations. The inherent differences in microbiota across the population, as well as the variations in methodology previously discussed, pose a significant challenge to the generation of microbiome-based clinical trials and therapeutics [35, 37]. Leveraging new technologies, such as artificial intelligence-based analysis techniques [35], or the development of larger scale studies could accelerate the translation of this new research field to clinical medicine.

## Conclusions

In conclusion, the field of microbiome research in the context of bladder cancer presents a multitude of challenges that need to be addressed to ensure the validity and clinical relevance of findings. The complexity of microbial communities, the low biomass of urinary samples, and the variability in methodologies across studies all contribute to the difficulties in interpreting and translating microbiome research into clinical applications. Future research efforts should focus on standardization, improved experimental models, and advanced analytical approaches to advance our understanding of the microbiome’s impact on bladder cancer and ultimately improve patient outcomes.

## Data Availability

No datasets were generated or analysed during the current study.
